# Supplementary material for: A stem group echinoderm from the basal Cambrian of China and the origins of Ambulacraria
Source: Nat Commun. 2019 Mar 25;10:1366. doi: 10.1038/s41467-019-09059-3 (PMC6433856; doi:10.1038/s41467-019-09059-3)
Supplement: Supplementary file 2 — Supplementary Information [file 41467_2019_9059_MOESM2_ESM.pdf]

Supplementary Information

A stem group echinoderm from the basal Cambrian of China and the origins of Ambulacraria

Topper et al.

This file comprises:

Supplementary Fig. 1: Locality and stratigraphy of the lower Cambrian (Fortunian) Yanjiahe Formation, Yichang, Hubei, China
Supplementary Fig. 2: *Yanjiahella biscarpa* from the lower Cambrian (Fortunian) Yanjiahe Formation. Specimen ELI-HS18.
Supplementary Fig. 3: *Yanjiahella biscarpa* from the lower Cambrian (Fortunian) Yanjiahe Formation. Specimen ELI-HS84.
Supplementary Fig. 4: *Yanjiahella biscarpa* from the lower Cambrian (Fortunian) Yanjiahe Formation, additional specimens.
Supplementary Fig. 5: *Yanjiahella biscarpa* from the lower Cambrian (Fortunian) Yanjiahe Formation, additional specimens.
Supplementary Fig. 6: Variations of tree topology of parsimony analyses under traditional heuristic search options with and without bootstrap resampling.
Supplementary Fig. 7: Variations of majority rule consensus trees of parsimony analyses under traditional heuristic search options.
Supplementary Fig. 8: Variations of tree topology of parsimony analyses under New Technology Search options with and without bootstrap resampling.
Supplementary Fig. 9: Strict consensus tree of a parsimony analysis under New Technology Search options.
Supplementary Note 1: Phylogenetic analyses including character list.
Supplementary Table 1: Data matrix used in the phylogenetic analyses.
Supplementary References

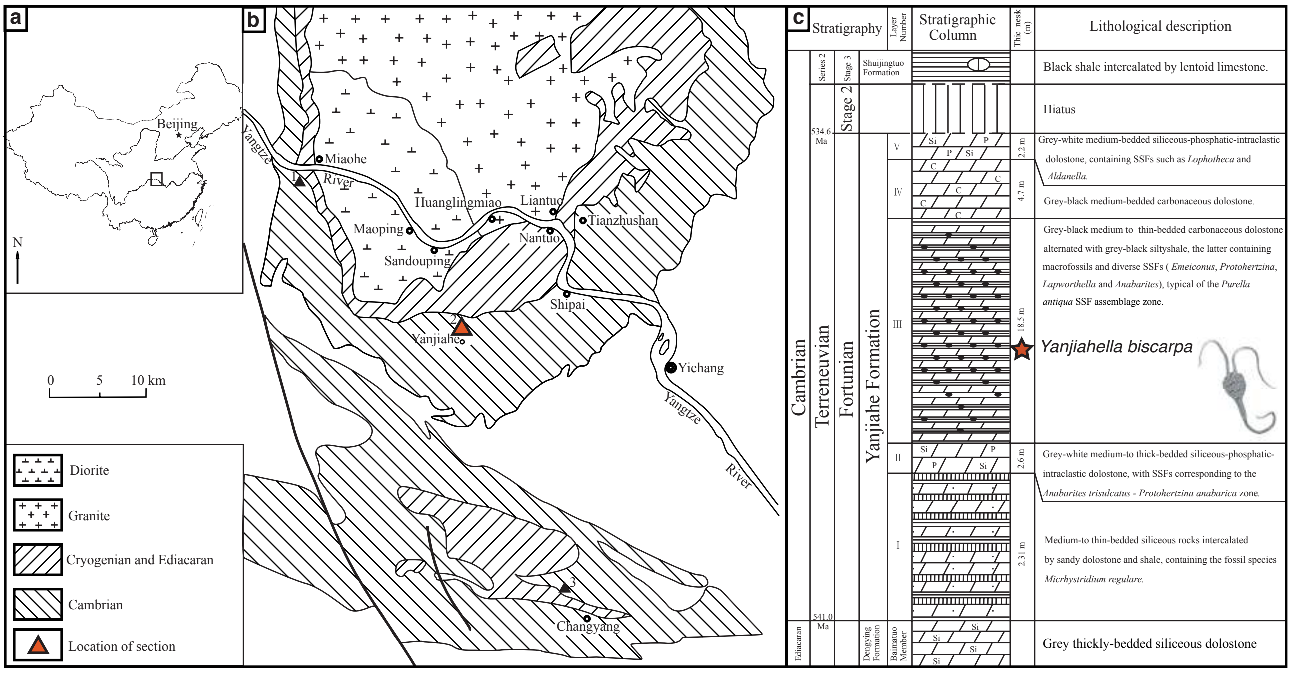

Supplementary Figure 1 Locality and stratigraphy of the lower Cambrian (Fortunian) Yanjiahe Formation, Yichang, Hubei, China. a. Map of the People's Republic of China, showing the position of the fossil locality (triangle) in Hubei province. b. Geological map of the Yanjiahe area, showing Cambrian outcrops. c. Stratigraphic sequence of lower Cambrian strata in the Gunziao section, indicating the horizon (star) where specimens of *Yanjiahella biscarpa* were collected that corresponds to the *Purella antiqua* shelly assemblage zone. The Fortunian age is also supported by chemostratigraphic correlation[1,2]. Figure modified from[1], request to reproduce figure granted by John Wiley & Sons (licence number 4525790680851). *Yanjiahella biscarpa* illustrated by Nobumichi Tamura.

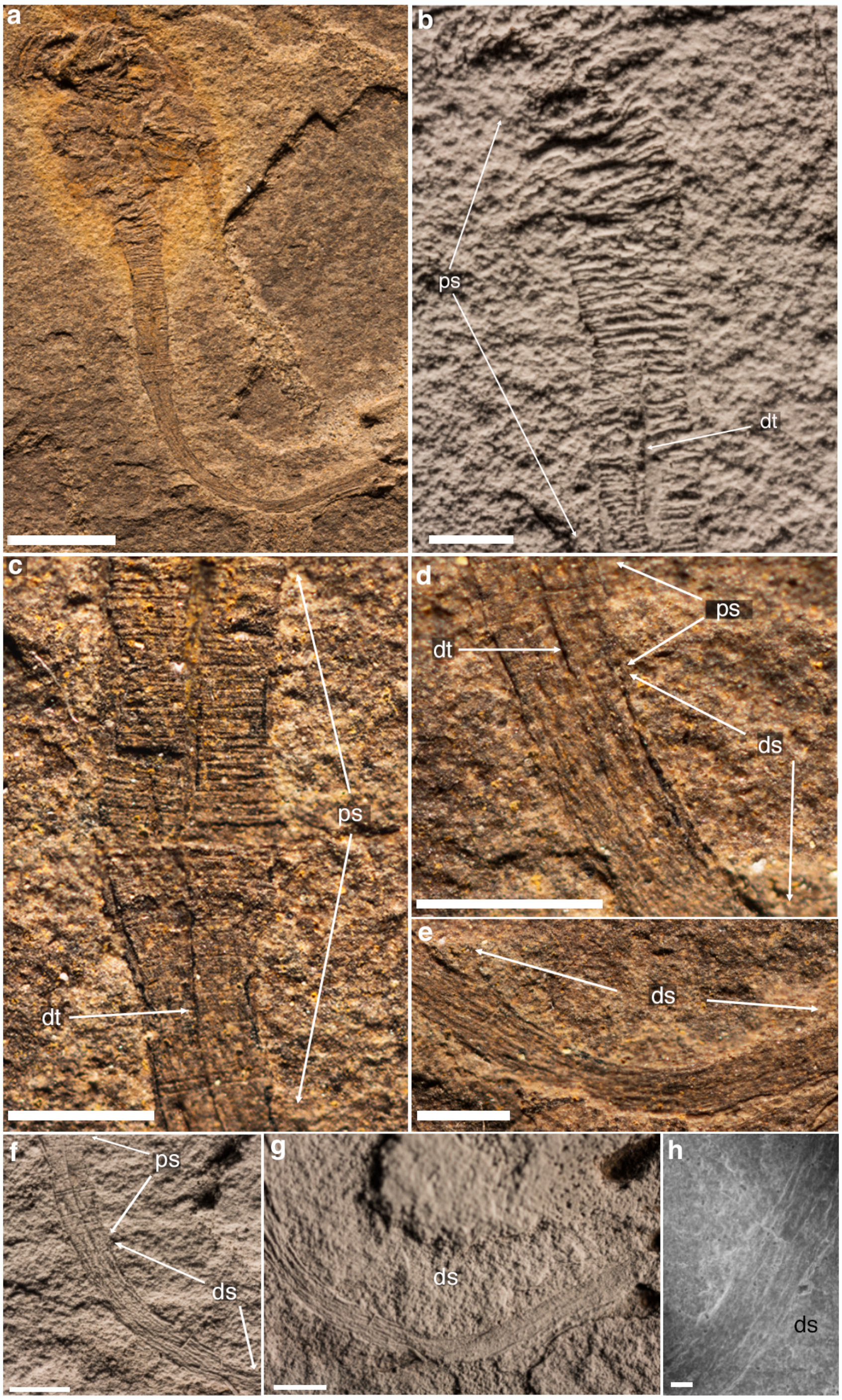

Supplementary Figure 2 *Yanjiahella biscarpa* from the lower Cambrian (Fortunian) Yanjiahe Formation. Specimen ELI-HS18. a. Specimen showing a plated theca, stalk and a feeding appendage. b. Proximal stalk showing transverse ridges, coated with ammonium chloride. c. Proximal stalk showing slight taper and a digestive tract. d. Transitional region of the proximal to distal stalk, note the change from transverse ridges to striations. e. Detail of the distal stalk with distinct striations visible. f. Proximal and distal zones of stalk coated with ammonium chloride. g. Detail of the distal stalk, coated with ammonium chloride. h. SEM image of distal stalk. Scale bars 5 mm (a), 1 mm (b-f), 0.5 mm (g) and 200 µm (h). Abbreviations: ds, distal stalk; dt, digestive tract; ps, proximal stalk.

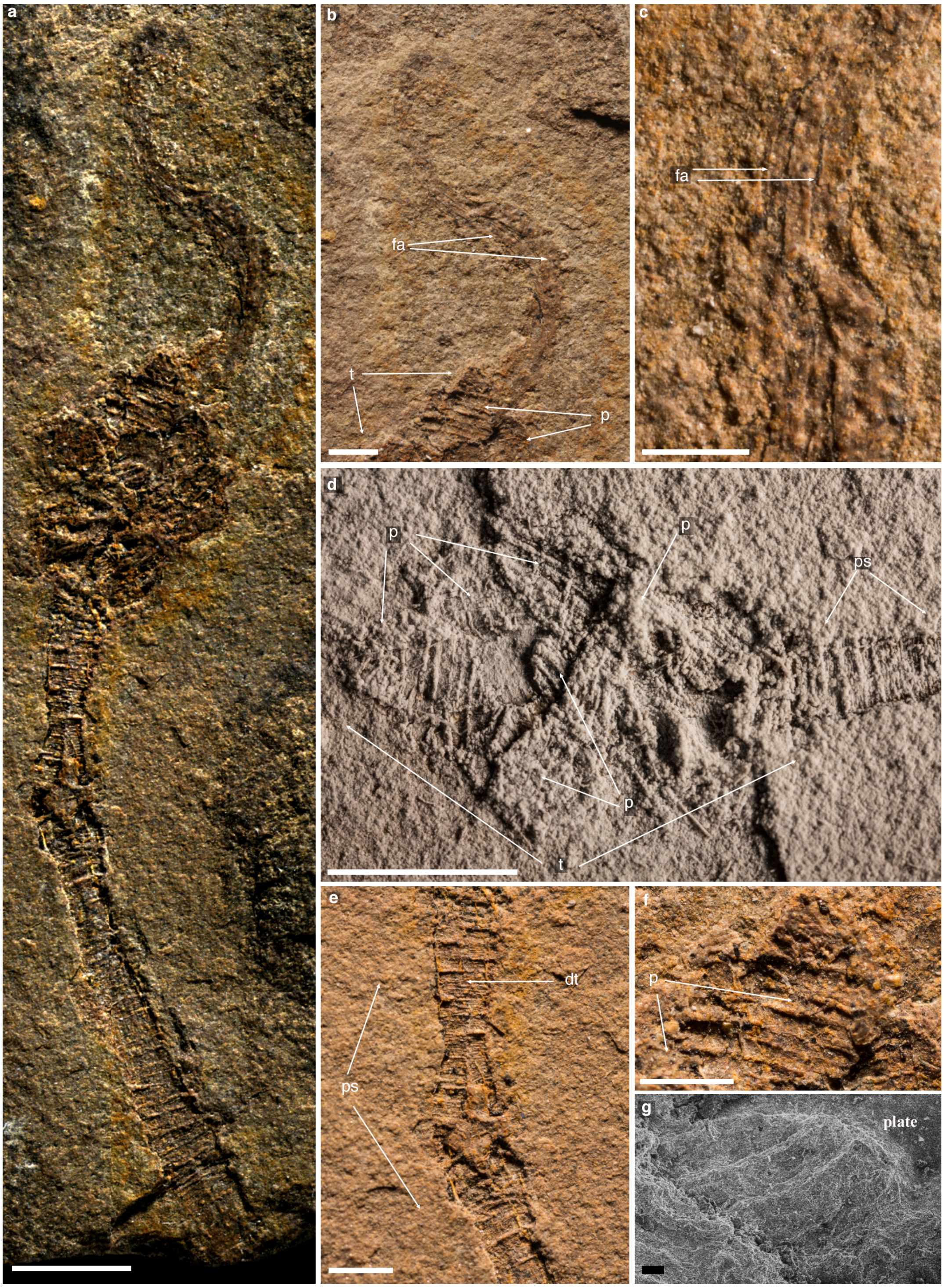

Supplementary Figure 3 *Yanjiahella biscarpa* from the lower Cambrian (Fortunian) Yanjiahe Formation. Specimen ELI-HS84. a. Stereo image of the specimen, showing plated theca, stalk and a pair of feeding appendages. b. Detail of juxtaposed feeding appendages. c. Detail of feeding appendages. d. Plated theca showing plates with parallel ornamentation. e. Muscular proximal stalk, showing distinct ridges and a median digestive tract. f. Plates showing parallel ornamentation. g. SEM image of a plate showing parallel ornamentation. Scale bars 3 mm (a, d), 1 mm (b, e, f), 0.5 mm (c) and 100 µm (g). Abbreviations: dt, digestive tract; fa, feeding appendage; p, plate; t, theca.

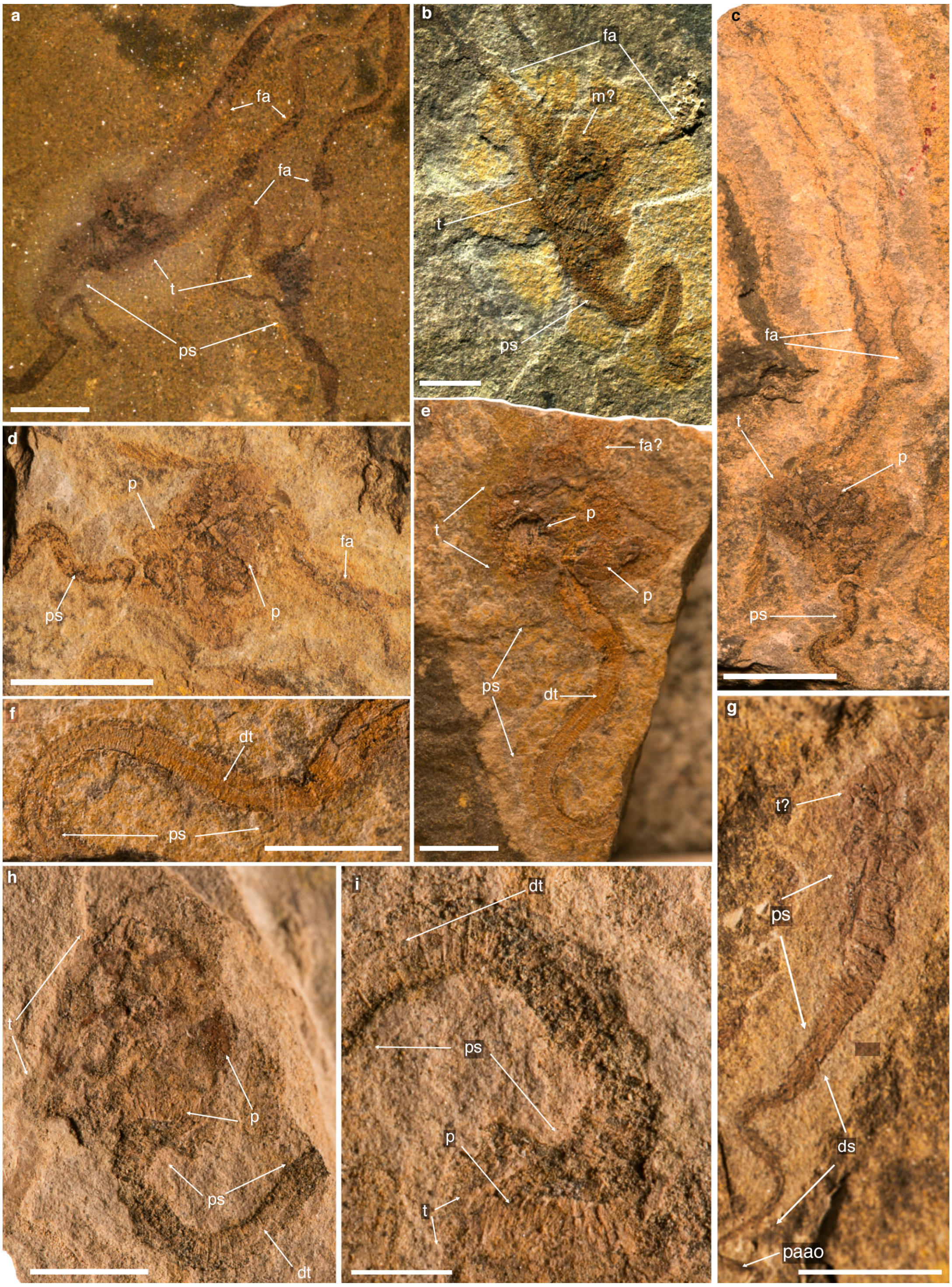

Supplementary Figure 4 *Yanjiahella biscarpa* from the lower Cambrian (Fortunian) Yanjiahe Formation. a. ELI-HS25B, two specimens, each with a pair of feeding appendages. b. Specimen ELI-HS205, Distorted theca with two feeding appendages. c-d. Specimen ELI-HS26B. c. Distorted theca, with two feeding appendages. d. Plated theca with plates showing parallel ornamentation. e-f. Specimen ELI-HS7B. e. Plated theca, stalk with transverse ridges and digestive tract. f. Stalk, with transverse ridges and digestive tract. g. Specimen ELI-HS69, Stalk with post anal anchoring organ and a possible distorted plated theca. h-i. Specimen ELI-G102. h. Specimen showing a plated theca and proximal stalk. i. Proximal stalk, showing transverse ridges and digestive tract. Scale bars 5 mm (a, b, e, f, g, h), 1 cm (c, d) and 2 mm (i). Abbreviations: ds, distal stalk; dt, digestive tract; fa, feeding appendage; m, mouth; p, plate; ps, proximal stalk; paao, post anal anchoring organ; t, theca.

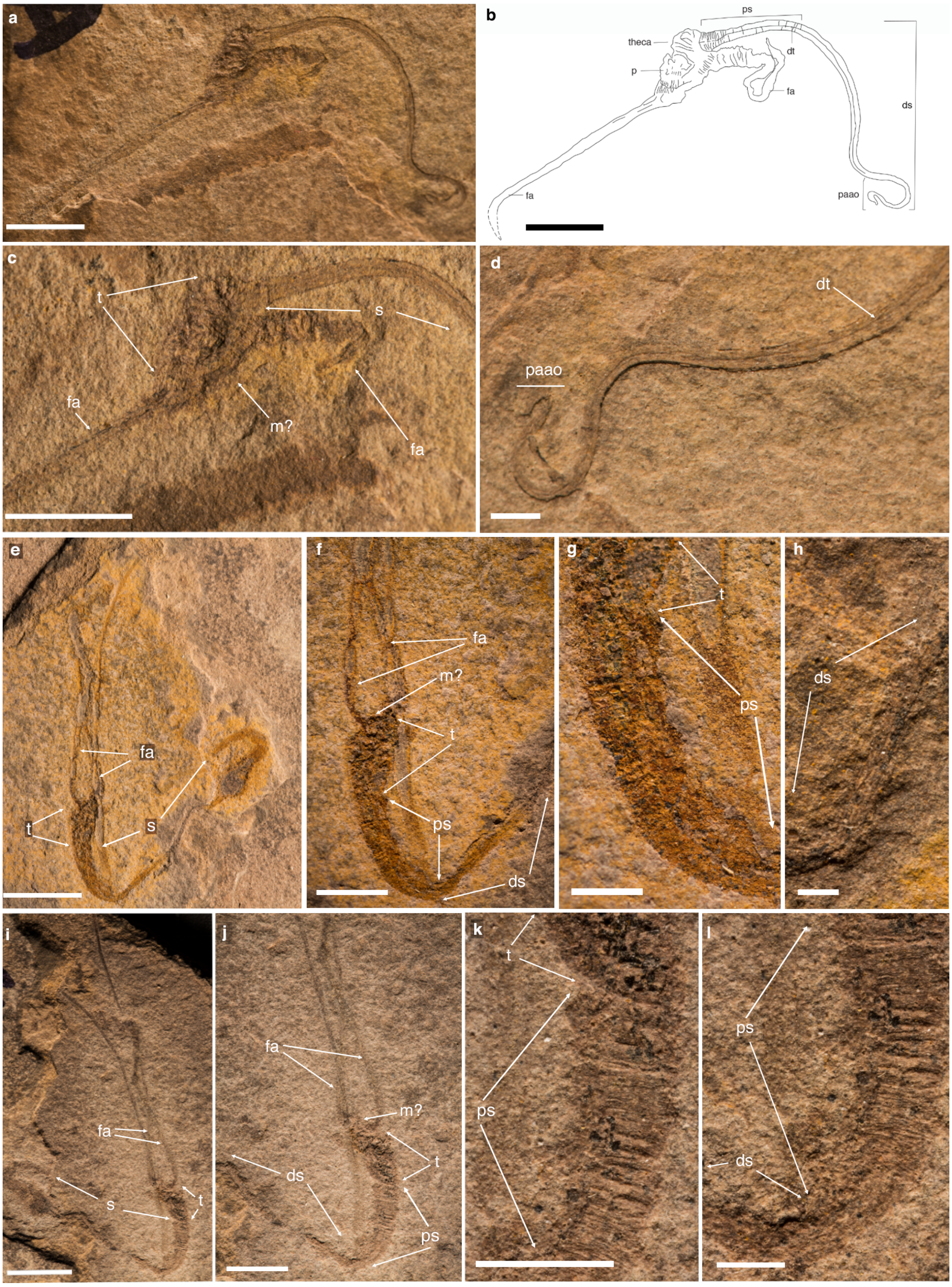

Supplementary Figure 5 *Yanjiahella biscarpa* from the lower Cambrian (Fortunian). a-d. Specimen ELI-HS99. a. Specimen showing a theca, muscular stalk and feeding appendages. b. Line drawing of specimen ELI-HS99. c. Theca, feeding appendages and stalk. d. Detail of distal stalk and post anal anchoring organ. e-h. Specimen HS998A (e-h; part) and Specimen HS998B (i-l; counterpart). e. Specimen with muscular stalk, theca and feeding appendages. f. Differentiated proximal and distal stalk. g. Detail of theca and proximal stalk. h. Detail of distal stalk with fine striations. i. Specimen showing a muscular stalk, theca and feeding appendages. j. Differentiated proximal and distal stalk. k. Detail of theca and proximal stalk. l. Detail of the proximal to distal stalk region. Scale bars 5 mm (a-c, f, j), 1 mm (d, l), 1 cm (e, i), 2 mm (g, k) and 0.5 mm (h). Abbreviations: ds, distal stalk; dt, digestive tract; fa, feeding appendage; m, mouth; p, plate; ps, proximal stalk; paao, post anal anchoring organ; s, muscular stalk; t, theca.

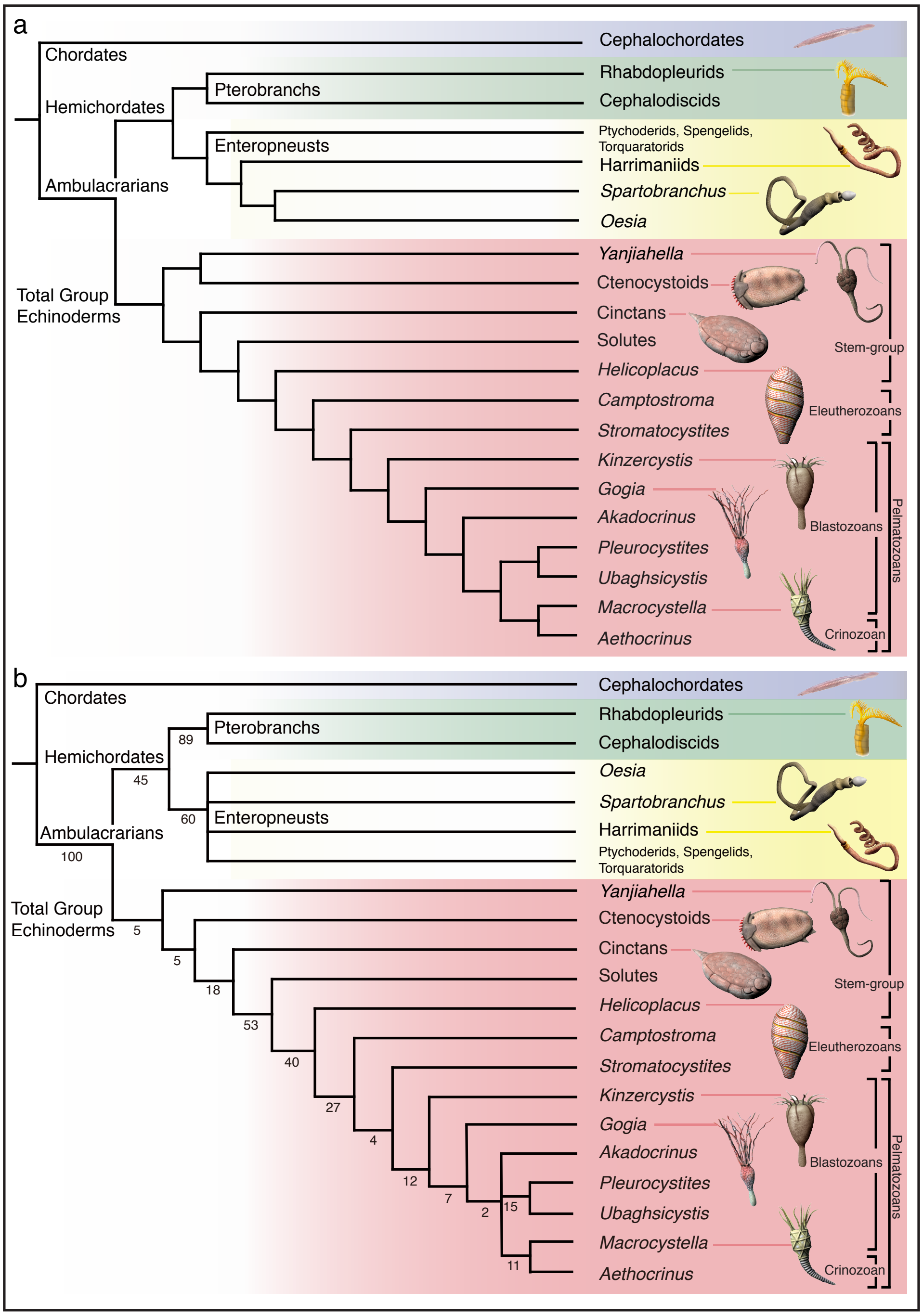

Supplementary Figure 6 Variations of tree topology of parsimony analyses under traditional heuristic search with 10, 000 replications and 10 trees saved per replication, without (a) and with (b) bootstrap resampling of 100 replicates. Characters unordered and with equal weights. 12 trees were found in both analyses with a total length of 70 steps (CI = 0.657, RI = 0.826). Without bootstrap resampling (a) *Yanjiahella* and the ctenocystoids form a sister group to the remaining total group echinoderms. Despite their association they share no synapomorphies. The tree obtained under bootstrap resampling (b) shows *Yanjiahella* positioned at the base of the total group echinoderms. The remainder of the echinoderm section of the tree is similar across both analyses, with the exception of some slight movement within the blastozoans. The enteropneusts (Cambrian and recent taxa) are grouped together in both analyses as a sister group to the pterobranchs, however without bootstrap resampling the Cambrian taxa are recovered as derived enteropneusts (a). Bootstrap values shown under each node. Taxa illustrated by Nobumichi Tamura.

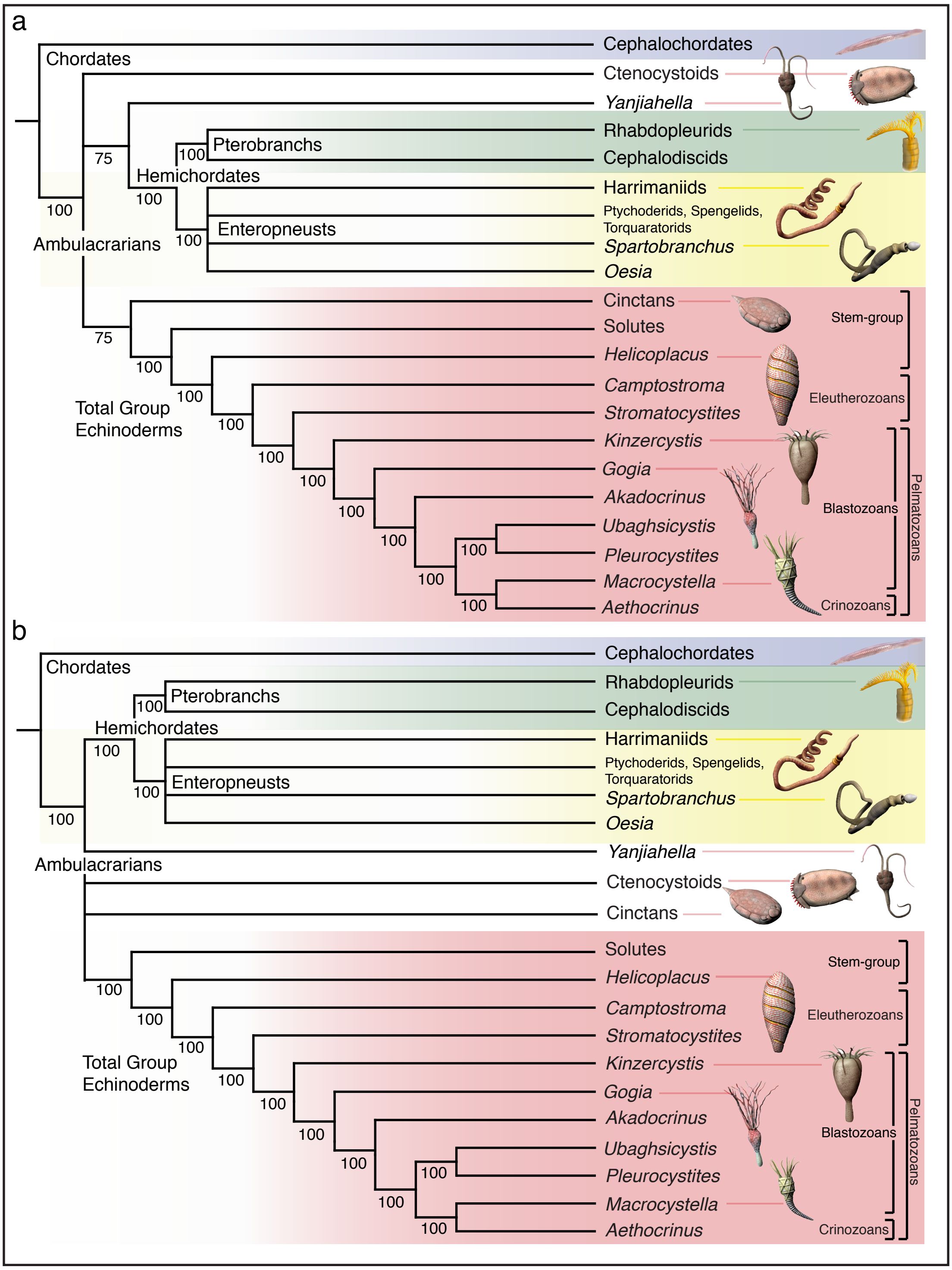

Supplementary Fig. 7 Variations of majority-rule consensus trees of parsimony analyses under traditional heuristic search options with 10, 000 replications and 10 trees saved per replication. (a) 50% majority-rule consensus tree, (b) 75% majority-rule consensus tree. Characters unordered and with equal weights. 12 trees were found in both analyses with a total length of 70 steps (CI = 0.657, RI = 0.826). The 50% majority-rule consensus trees shows ctenocystoids as basal ambulacrarians and *Yanjiahella* as a basal hemichordate. The 75% majority-rule consensus tree instead shows an unresolved basal ambulacrarian radiation recognizing *Yanjiahella*, ctenocystoids and cinctans in a polytomy along with the hemichordates and the remaining total group echinoderms. Taxa illustrated by Nobumichi Tamura.

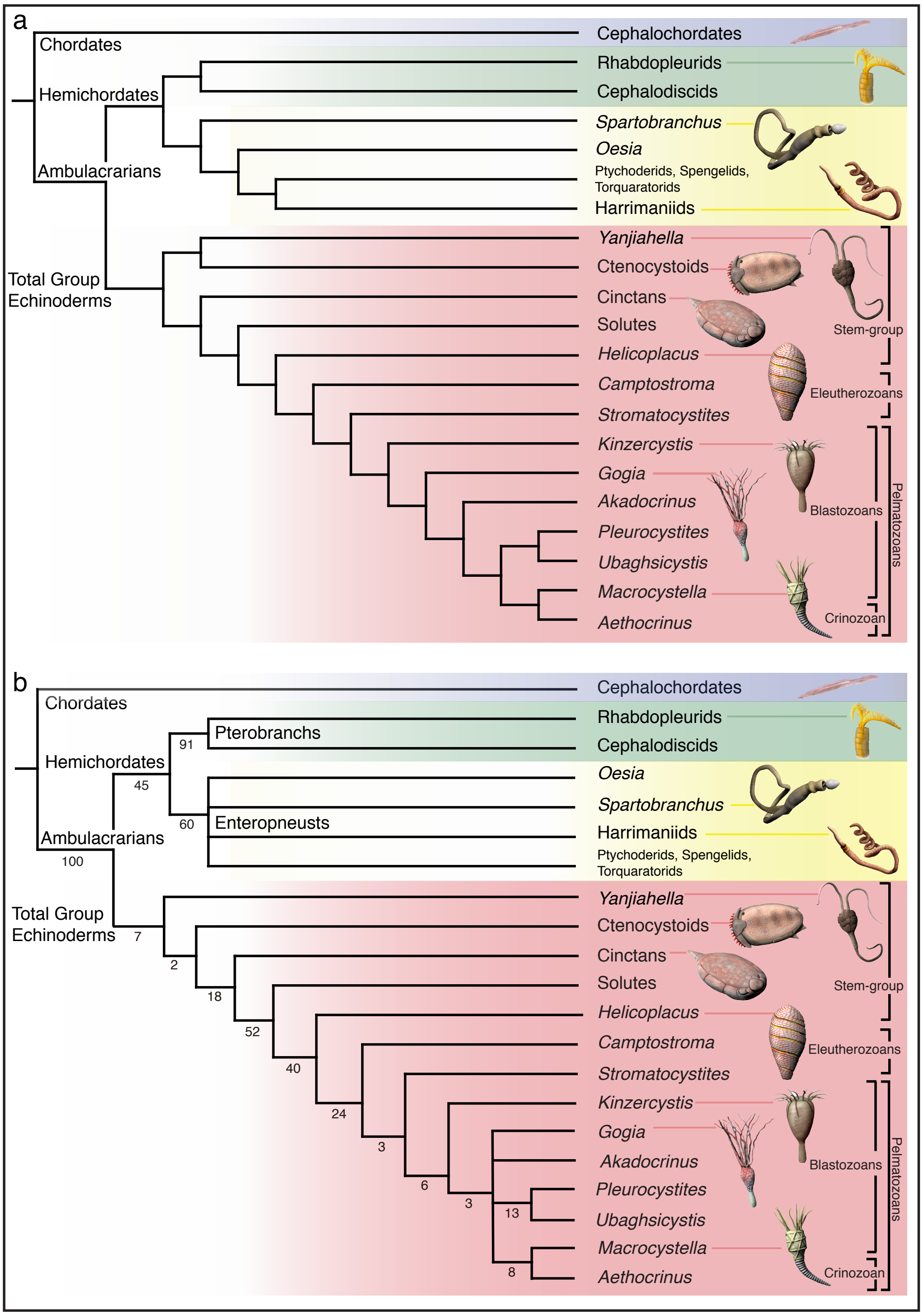

Supplementary Figure 8 Variations of tree topology of parsimony analyses under New Technology Search using Sectorial Search and Tree Fusing Options, without (a) and with (b) bootstrap resampling of 100 replicates. Five trees were retained with a total length of 70 steps (CI = 0.657, RI = 0.826). Comparisons between the analyses show only a slight change in tree topology and are very similar to the tree topology obtained using traditional heuristic search methods (Supplementary Figure 6). Without bootstrap resampling (a**)** *Yanjiahella* and the ctenocystoids are once again a sister group to the remaining total group echinoderms included in the analysis. The Cambrian taxa *Spartobranchus* and *Oesia* are however recovered as basal enteropneusts, a notable reversal from the topology of the tree achieved through traditional search methods (Supplementary Figure 6a). The tree obtained with bootstrap resampling is very similar to the tree shown in the main text (Fig. 3). Bootstrap values shown under each node. Taxa illustrated by Nobumichi Tamura.

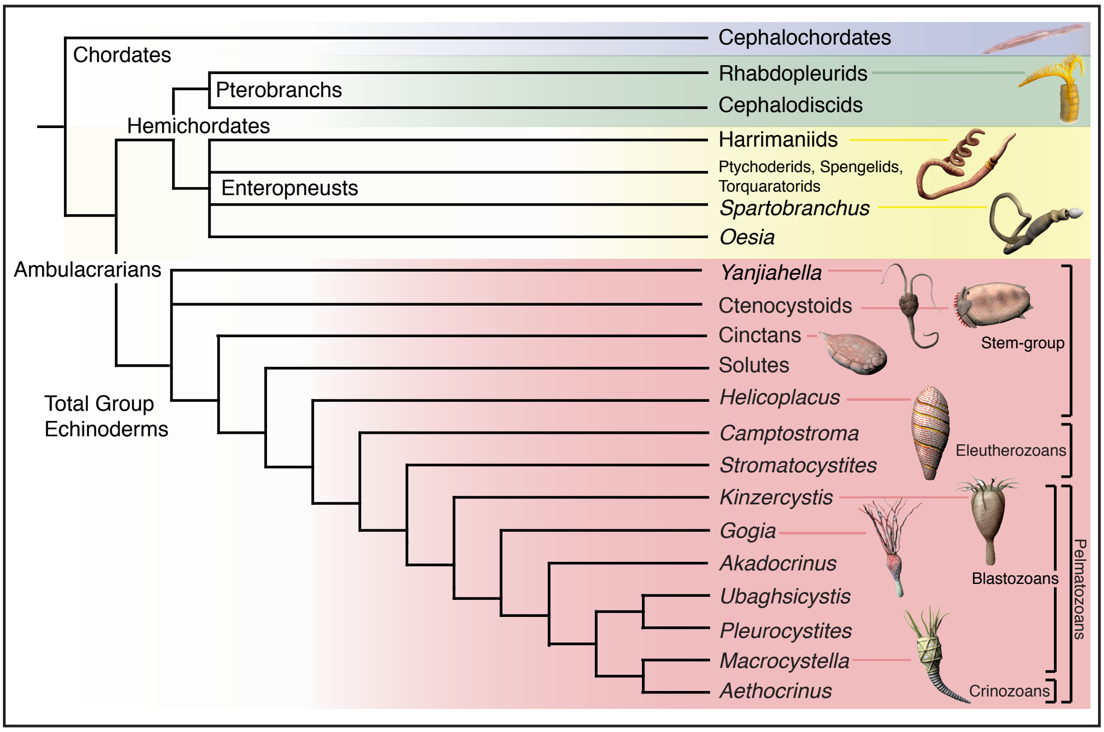

Supplementary Fig. 9 Strict consensus tree of parsimony analysis under New Technology Search options using Sectorial Search and Tree Fusing Options. Two trees were retained and best score was hit 1 time. The strict consensus tree is similar to the previously shown trees obtained using New Technology Search options. *Yanjiahella* and the ctenocystoids are however, unresolved as basal total group echinoderms, rather than a sister group to the remaining total group echinoderms (Supplementary Figure 8a). Taxa illustrated by Nobumichi Tamura.

Supplementary Note 1: Phylogenetic analyses.

Phylogenetic analysis: The phylogenetic analysis was conducted using TNT1.1[3] using, Traditional Heuristic Search, New Technology Search and Implicit Enumeration (details in main text and figure captions).

Taxa: Twenty-one taxa have been included in our analysis, incorporating fourteen total group Echinoderms (including *Yanjianella biscarpa*), four enteropneust taxa (including both *Spartobranchus tenuis* and *Oesia disjuncta* from the Cambrian Burgess Shale Lagerstätte), two pterobranch taxa and the Cephalochordates (used as the outgroup). The taxa encompass all the major echinoderm body plans present in the early to middle part of the Cambrian, such as the bilateral symmetrical ctenocystoids (coded from *Courtessolea*) and the asymmetrical cinctans (coded from *Asturicystis)* and solutes (coded from *Castericystis*). However, we have excluded the stylophorans, whose interpretation remains highly contentious[4-9]. We included *Pleurocystites* (an Early Ordovician to Middle Devonian glyptocystitid rhombiferan) and its probable stem-relative *Ubaghsicystis* (Cambrian Series 3, Wuliuan), as both taxa share some similarities with *Yanjianella* (two arms, globular to flattened theca, probable muscular appendage)[10]. Different pelmatozoan representatives have been added to the study such as the Ordovician *Macrocystella*[11] and the crinozoan *Aethocrinus*[12]. The enteropneust taxa that we have included have been broadly arranged into two groups. The families Ptychoderidae, Spengelidae and Torquaratoridae (labeled 'Enteropneusts' in the matrix below) are one group and the Harrimaniidae are another. The four families of Enteropneusta are only known from living taxa and their inter-relationships have been the subject of many recent morphological and molecular studies[13-17]. The relationships between living enteropneust families is not the focus of this study and as we could provide no further morphological details than that which has already been presented in previous studies[13] we have grouped the families together. The Harrimanidae is separated from the remaining three families of enteropneust because they are generally considered to be basal among the Enteropneusta[15]. *Spartobranchus tenuis* and *Oesia disjuncta* represent some of the oldest recognized members of the Hemichordata[18-20] and they have been included herein to better test possible hemichordate affinities of the Cambrian *Yanjianella*. Cephalochordates are chosen as the outgroup taxa, as the Chordata represent a sister group to the Ambulacraria and cephalochordates are commonly recognized as a basal member of the Chordate clade [17, 21-22].

Characters: A total of 42 phylogenetically informative characters were included in the analysis of 21 taxa. This character list has been modified from previous published datasets[23-24] and adapted for the purpose of this study with additional taxa and characters. Where possible, characters have been coded as presence/absence of determinate homologous features. Character descriptions are as follows:

1. **Stomochord present: no (0); yes (1)**
2. **Possession of a dorsal collar nerve cord (neurocord): no (0); yes (1)**
   Chordates possess a dorsal hollow nerve cord and hemichordates possess a neurocord. Although the homology of these structures is unclear[25], the collar cord of enteropneusts has been considered by some to be homologous with the neurulation in chordates[26]. This discussion appears far from settled, however for the purpose of this analysis we have united them here under a single character state.
3. **Body plan: bilaterial (0); asymmetric (1); radial (2)**
   The body plan of cinctans varies from asymmetrical to nearly bilaterally symmetrical, with symmetry expressed

in the shape of the theca and the size/ number of the anterior feeding grooves[27-28]. This is most likely related to their asymmetric development of the hydrocoel during their metamorphosis from larva to adult[27-28]. As such, cinctans have been here coded as exhibiting an asymmetric body plan.

4. **Pentaradial symmetry developed: no (0) yes (1)**
   Of those echinoderm forms with a radial body plan *Helicoplacus* does not show pentaradial symmetry, instead showing three-fold symmetry[23].
5. **Anterior-posterior (mouth-anus) axis: no (0); yes (1)**
6. **Possession of symmetrical mesocoels (hydrocoels) as adult: no (0); yes (1)**
   Hemichordates and echinoderms have a homologous mesocoel (hydrocoel in echinoderms). This is paired and symmetrical in Hemichordates. Most echinoderms have an asymmetric hydrocoelom, the right mesocoel regressing (to complete degeneration in crown groups). Even if a bilateral symmetry is seen in *Yanjianella biscarpa* and the ctenocystoids the presence of a paired, symmetrical mesocoel can only be at best suggested. As such, the presence of paired hydrocoel in the apparently symmetrical *Yanjianella biscarpa* cannot be accurately assessed (scored as ?).
7. **Torsion resulting in vertical stacking of body coeloms and complete suppression of right hydrocoel development in adult form: no (0); yes (1)**
   Echinoderms have a unique ontogeny where the organism undergoes a phase of torsion in development that rotates the axis of the developing adult [29]. Although no ontogenetic information regarding *Yanjiahella biscarpa* is known, the clear Anterior-Posterior (A-P) axis in all specimens, suggests that the taxon did not undergo torsion. The A-P axis in solutes has been used to support a pre-torsion history of the echinoderms [29] and it has been suggested that the helicoplacoids represent the earliest echinoderms to have undergone complete torsion, as both dorsoventral and left-right body axes are lost and the hydrocoel displays a radial organization[29]. We follow this interpretation herein and have coded all bilateral and asymmetric total group echinoderm taxa (*Yanjiahella biscarpa*, ctenocystoids, cinctans and solutes) as not having undergone complete torsion.
8. **Pharyngeal gill slits and/or pore(s) present: no (0); yes (1)**
   A recent study[21] regarding gill development genes, supported the homology of echinoderm and hemichordates gills. Such gills slits and, or pores have been described in stylophoran echinoderms (not included in this analysis) and there are strong morphological indications that such structures are present in cinctans and ctenocystoids[30-31] (character state for both taxa coded here as present). Poor plate preservation does not allow an accurate assessment regarding the presence of gills/pore in *Yanjianella biscarpa.* Thecal plates of *Yanjianella biscarpa* might not have abutted during life so it is possible that gills may have opened between the plates, within the soft tegument (consequently character state is questioned herein).
9. **Gill slits have secondary (or tongue) bars: no (0); yes (1)**
   Gill bars are a morphological feature shared by enteropneusts, including the two Cambrian taxa (*Oesia* and *Spartobranchus*) where such structures are among the most common and well-preserved internal features[18-20].
10. **Possession of pharyngeal (branchial) region: no (0); yes (1)**
11. **Proboscis bearing (including cephalic shield): no (0); yes (1)**
12. **Proboscis shape: acorn-shaped (0); flattened (1)**
13. **Possession of a nuchal skeleton: no (0); yes (1)**
   This collagenous structure underlies the stomochord in the proboscis of the majority of enteropneusts, being reduced or absent in the deep-sea torquaratoids. This structure has also been documented from both Cambrian

enteropneust taxa[18-20].

14. **Possession of a muscular trunk, tail or stalk: no (0); yes (1)**

    All hemichordates have a muscular trunk (enteropneust) or stalk (pterobranch). Among included echinoderms, muscular appendages have been described in Pleurocystitiids[10], and also suggested in solutes (e.g. [32] and references therein; character state questioned herein). The stalk of *Yanjianella biscarpa* is interpreted herein to be muscular (see main text).

15. **Possession of ossicles (plates) embedded in the body wall: no (0); yes (1)**
16. **Possession of calcitic skeleton made of stereom: no (0); yes (1)**

    The poor preservation of *Yanjianella biscarpa* thecal plates does not allow stereom microstructure to be observed. Therefore, this character state is questioned, although stereom is interpreted herein to have been present in *Y. biscarpa*.

17. **Marginal frame present: no (0); yes (1)**

    Only the ctenocystoids and cinctans in this analysis have a body form framed by broad marginal ossicles.

18. **Ambulacra present: no (0); yes (1)**

    Evidence for flooring/cover plates associated with the water vascular system is found in all echinoderms here, except cinctans, ctenocystoids and *Yanjianella biscarpa*.

19. **Ambulacra: straight (0); curved and spiralling distally (1)**

    Pelmatozoans without ambulacra forming an integral part of the theca are scored as inapplicable.

20. **Ambulacral flooring plates forming an integral part of the theca: no (0); yes (1)**
21. **Interambulacral zones with folded concertina-like structure (0); simple pavements of plates (1)**
22. **Stalk or stem: absent (0); present (1)**
23. **Stalk or stem: non plated (0); plated (1)**
24. **Plated stalk or stem: not clearly separated from "cup" (0), clearly differentiated structure (1)**
25. **Stalk differentiated in proximal and distal regions: no (0); yes, major change in diameter and, or plate construction between proximal and distal parts (1)**
26. **Distal stalk plating: polyplated and imbricate elements (0); holomeric (1)**
27. **Proximal stalk plating: polyplated and imbricate elements (0); holomeric (1); polymeric (2)**
28. **Proximal stalk plating: with articulating ring flange (pleurocystitid or glyptocystitid like): no (0), yes (1)**
29. **Theca: sac-like (0); flattened (1)**
30. **Flooring plates of theca perforate: no (0); yes (1)**

    Perforation of flooring plates connects the ambulacra groove to the interior of the theca. Scored as inapplicable for echinoderms lacking flooring plates (most blastozoans).

31. **Theca organized in a small number of standardized circlets: no, (e.g. *Gogia*) (0), yes (e.g. *Macrocystella*) (1)**
32. **Oral region of theca raised as a spout and bearing free arms: no (0); yes (1).**
33. **Position of mouth in axial zone: lateral (0); central and apical (1)**

Scored as inapplicable where no axial zone/ambulacral zone is recognizable (following [23])

34. **Anus or periproct position: within the oral zone (0), lateral (1), posterior (2)**
35. **Epispires present: no (0); yes (1)**
36. **Respiratory rhombs: absent (0); present (1)**
37. **Respiratory folds on thecal plates: absent or very reduced (0); present (1)**
38. **Free feeding appendage(s): absent (0); yes, soft (1), yes skeletonized (2)**

39. **Skeletonized free feeding appendage(s): with fine biserial flooring elements (0); uniserial extension of the theca (1)**

40. **Free feeding appendages isotomously branched: no (0); yes (1)**

41. **Secretion of an external tubular structure (tube-dweller): no (0); yes (1)**

The Cambrian hemichordate taxa are tubiculous organisms[18-20]. Even if all four families of extant enteropneust can secrete mucous “tubes” when stressed, this structure is not homologous to those of their Cambrian relatives[19]. No equivalent is known in echinoderms. There is no conclusive evidence to suggest that this character was present in *Yanjianella biscarpa*, which instead possesses a skeletonized tubular body chamber, and a weakly skeletonized stalk, and is considered herein as such.

42. **Life behaviour: Colonial (0); Solitary (1)**

Supplementary Table 1: Data matrix

	1	2	3	4	5	6	7	8	9	10	11	12	13	14	15	16	17	18	19	20	21
Cephalochordates	0	1	1	0	1	?	0	1	1	0	0	-	0	0	0	0	-	-	-	-	-
Harimaniids	1	1	0	0	1	1	0	1	1	1	1	0	1	1	0	0	-	-	-	-	-
Enteropneusts	1	1	0	0	1	1	0	1	1	1	1	0	1	1	0	0	-	-	-	-	-
Spartobranchus	1	1	0	0	1	1	0	1	1	1	1	0	1	1	0	0	-	-	-	-	-
Oesia	1	1	0	0	1	1	0	1	1	1	1	0	1	1	0	0	-	-	-	-	-
Rhabdopleurids	1	0	0	0	0	1	0	0	-	0	1	1	0	1	0	0	-	-	-	-	-
Cephalodiscids	1	0	0	0	0	1	0	1	0	0	1	1	0	1	0	0	-	-	-	-	-
Yanjiahella	0	0	0	0	1	?	0	?	?	0	0	-	0	1	1	?	0	0	-	-	-
Ctenocystoids	0	0	0	0	1	?	0	1	?	0	0	-	0	0	1	1	1	0	-	-	-
Cinctans	0	0	1	0	0	0	0	1	?	0	0	-	0	0	1	1	1	0	-	-	-
Solutes	0	0	1	0	0	0	0	0	-	0	0	-	0	?	1	1	0	1	-	0	-
Helicoplacus	0	0	2	0	0	0	1	0	-	0	0	-	0	0	1	1	0	1	1	1	1
Kinzercystis	0	0	2	1	0	0	1	0	-	0	0	-	0	0	1	1	0	1	0	1	0
Gogia	0	0	2	1	0	0	1	0	-	0	0	-	0	0	1	1	0	1	-	0	0
Akadocrinus	0	0	2	1	0	0	1	0	-	0	0	-	0	0	1	1	0	1	?	?	0
Macrocystella	0	0	2	1	0	0	1	0	-	0	0	-	0	0	1	1	0	1	-	0	0
Ubaghsicystis	0	0	2	1	0	0	1	0	-	0	0	-	0	0	1	1	0	1	-	0	0
Pleurocystites	0	0	2	1	0	0	1	0	-	0	0	-	0	1	1	1	0	1	-	0	0
Aethocrinus	0	0	2	1	0	0	1	0	-	0	0	-	0	0	1	1	0	1	0	0	0
Stromatocystites	0	0	2	1	0	0	1	0	-	0	0	-	0	0	1	1	0	1	0	1	0
Camptostroma	0	0	2	1	0	0	?	0	-	0	0	-	0	0	1	1	0	1	1	1	0
	**22**	**23**	**24**	**25**	**26**	**27**	**28**	**29**	**30**	**31**	**32**	**33**	**34**	**35**	**36**	**37**	**38**	**39**	**40**	**41**	**42**
Cephalochordates	0	-	-	-	-	-	-	-	-	-	-	-	2	-	-	-	-	-	-	0	1
Harimaniids	0	-	-	-	-	-	-	-	-	-	-	-	2	-	-	-	-	-	-	0	1
Enteropneusts	0	-	-	-	-	-	-	-	-	-	-	-	2	-	-	-	-	-	-	0	1
Spartobranchus	0	-	-	-	-	-	-	-	-	-	-	-	2	-	-	-	-	-	-	1	1
Oesia	0	-	-	-	-	-	-	-	-	-	-	-	2	-	-	-	-	-	-	1	1
Rhabdopleurids	0	-	-	-	-	-	-	-	-	-	-	-	0	-	-	-	-	-	-	1	0
Cephalodiscids	0	-	-	-	-	-	-	-	-	-	-	-	0	-	-	-	-	-	-	1	0
Yanjiahella	1	0	1	1	-	-	-	0	-	?	0	-	2	0	0	0	1	-	0	?	1
Ctenocystoids	0	-	-	-	-	-	-	1	-	0	-	-	2	0	0	0	0	-	0	-	1
Cinctans	0	-	-	-	-	-	-	1	-	0	-	-	1	0	0	0	0	-	0	-	1
Solutes	1	1	0	1	0	0	0	0	-	0	0	0	1	0	0	0	2	0	0	-	1
Helicoplacus	0	-	-	-	-	-	-	0	1	0	-	0	0	0	0	0	0	-	0	-	1
Kinzercystis	1	1	0	0	0	0	0	0	0	0	0	1	0	1	0	0	2	0	0	-	1
Gogia	1	1	1	0	0	0	0	0	-	0	0	1	1	1	0	0	2	0	0	-	1
Akadocrinus	1	1	1	0	1	1	0	0	-	0	0	1	?	1	0	0	2	0	0	-	1
Macrocystella	1	1	1	1	1	1	1	0	-	1	0	1	1	0	0	1	2	0	0	-	1
Ubaghsicystis	1	1	1	0	1	1	0	1	-	0	1	1	1	0	0	1	2	0	0	-	1
Pleurocystites	1	1	1	1	1	1	1	1	-	0	1	1	1	0	1	0	2	0	0	-	1
Aethocrinus	1	1	1	0	0	2	0	0	-	1	0	1	0	0	0	1	2	1	1	-	1
Stromatocystites	0	-	-	-	-	-	-	0	1	0	-	1	0	1	0	0	0	-	0	-	1
Camptostroma	0	-	-	-	-	-	-	0	1	0	-	1	0	1	0	0	0	-	0	-	1

Supplementary References

1. Guo, J., Yong, L.I., Han, H., Ou, Q. Zhou, J. & Zheng, Y. New Macroscopic Problematic Fossil from the Early Cambrian Yanjiahe Biota, Yichang, Hubei, China. *Acta Geol. Sin.* **86**, 791-198 (2012).

2. Ishikawa, T. *et al.* Carbon isotope chemostratigraphy of a Precambrian/Cambrian boundary section in the Three Gorge area, South China: prominent global-scale isotope excursions just before the Cambrian Explosion. *Gond. Res.* **14,** 193-208 (2008).

3. Goloboff, Pablo A., James S. Farris, & Nixon, K.C. TNT, a free program for phylogenetic analysis. *Cladistics* **24**, 774-786 (2008).

4. Ubaghs, G. Stylophora. In: Treatise on Invertebrate Paleontology. Part S, Echinodemata 1 (2) (ed. R. C. Moore). The University of Kansas and the Geological Society of America, S495–S565 (1968).

5. Jefferies, R. P. S., Brown, N. & Daley, P. The early phylogeny of chordates and echinoderms and the origin of chordate left-right asymmetry and bilateral symmetry. *Acta Zoologica* **77**, 101-122 (1996).

6. David, B., Lefebvre, B., Mooi, R. & Parsley, R. Are homalozoans echinoderms? An answer from the extraxial-axial theory. *Paleobiology* **26**, 529-555 (2000).

7. Clausen, S. & Smith, A. B. Palaeoanatomy and biological affinities of a Cambrian deuterostome (Stylophora). *Nature* **438**, 351-354 (2005).

8. Rahman, I. A., Jefferies, R.P.S., Südkamp, W.H., Smith, R.D.A. Ichnological insights into mitrate palaeobiology. *Palaeontology* **52**, 127-238 (2009).

9. Lefebvre, B. *et al*. Exceptionally preserved soft parts in fossils from the Lower Ordovician of Morocco clarify stylophoran affinities within basal deuterostomes. *Geobois*. https://doi.org/10.1016/j.geobios.2018.11.001 (in press).

10. Gorzelak, P, Zamora, S.Understanding form and function of the stem in early flattened echinoderms (pleurocystitids) using a microstructural approach. *PeerJ* 4:e1820. https://doi.org/10.7717/peerj.1820 (2016).

11. Paul, C.R.C. *Macrocystella* Callaway, the earliest glyptocystitid cystoid. *Palaeontology* **11**, 580-600 (1968).

12. Lefebvre et al. Palaeoecological aspects of the diversification of echinoderms in the Lower Ordovician of central Anti-Atlas, Morocco. *Pal. Pal. Pal.* **460**, 97-121 (2016).

13. Cameron, C.B. A phylogeny of the hemichordates based on morphological characters. *Can. J. Zool.* **83**, 196-215 (2005).

14. Cannon, J. T., Rychel, A. L., Eccleston, H., Halanych, K. M. & Swalla, B. J. Molecular phylogeny of Hemichordata, with updated status of deep-sea enteropneusts. *Mol. Phylogenet. Evol*. **52**, 17–24 (2009).

15. Cannon, J.T., Kocot, K.M., Waits, D.S., Weese, D.A., Swalla, B.J., Santos, S.R. & Halanych, K.M. Phylogenomic resolution of the hemichordate and echinoderm clade. *Current Biology* **24**, 2827-2832 (2014).

16. Halanych, K.M., Cannon, J.T., Mahon, A.R., Swalla, B.J. & Smith, C.R. Modern Antarctic acorn worms form tubes. *Nature Comm. 4*, 2738 (2013).

17. Tassia M.G., Cannon J.T., Konikoff C.E., Shenkar N., Halanych K.M., Swalla B.J. The Global Diversity of Hemichordata. *PLoS One* **11**, e0162564 (2016).

18. Caron, J.B., Morris, S.C. & Cameron, C.B., 2013. Tubicolous enteropneusts from the Cambrian period. *Nature* **495**, 503 (2013).

19. Nanglu, K., Caron, J. -B. & Cameron, C. B. Using experimental decay of modern forms to reconstruct the early evolution and morphology of fossil enteropneusts. *Paleobiology* **41**, 460-478 (2015).

20. Nanglu, K., Caron, J. -B., Conway Morris, S. & Cameron, C. B. Cambrian suspension-feeding tubicolous hemichordates. *BMC Biol.* **14**, 56 (2016).

21. Simakov, O. *et al*. Hemichordate genomes and deuterostome origins. *Nature* **527**, 459-465 (2015).

22. Cannon, J.T., Vellutini, B.C., Smith, J., Ronquist, F., Jondelius, U. & Hejnol, A. Xenacoelomorpha is the sister group to Nephrozoa. *Nature* **530**, 89-93 (2016).

23. Smith, A.B. & Zamora, S. Cambrian spiral-plated echinoderms from Gondwana reveal the earliest pentaradial body plan. *Proc. R. Soc. B* **280**, 20131197 (2013).

24. Zamora, S. & Smith, A.B. Cambrian stalked echinoderms show unexpected plasticity of arm construction. *Proc. R. Soc. B* **279**, 293-298 (2012).

25. Ruppert, E.E. Key characters uniting hemichordates and chordates: homologies or homoplasies?. *Can. J. Zool.* **83**, 8-23 (2005).

26. Kaul-Strehlow, S. & Stach, T. A detailed description of the development of the hemichordate *Saccoglossus kowalevskii* using SEM, TEM, Histology and 3D-reconstructions. *Front. Zool*. **10**, 53 (2013).

27. Rahman, I.A. & Zamora, S. The oldest cinctan carpod (stem-group Echinodermata), and the evolution of the water vascular system. *Zool. J. Linnean Soc*. **157**, 420-432 (2009).

28. Zamora, S. & Rahman, I. A. Deciphering the early evolution of echinoderms with Cambrian fossils. *Palaeontology*, **57**, 1105-1119 (2014).

29. Smith, A. B. Deuterostomes in a twist: the origins of a radicial new body plan. *Evol. & Develop*. **10**, 493-503 (2008).

30 – Rahman, I.A. & Clausen, S. Re-evaluating the palaeobiology and affinities of the Ctenocystoidea (Echinodermata). *J. System. Palaeo.* **7**, 413-426 (2009).

31– Rahman, I.A., Zamora, S., Falkingham, P.L. & Phillips, J.C. Cambrian cinctan echinoderms shed light on feeding in the ancestral deuterostome. *Proc. R. Soc. B* **282**, p.20151964 (2015).

32. Rahman, I.A. & Lintz, H. *Dehmicystis globulus*, an enigmatic solute (Echinodermata) from the Lower Devonian Hunsrück Slate, Germany. *Paläont. Zeit.* **86**, 59-70 (2012).
